# Account of Diseases in an Eastern District from the 20th of January to the 20th of February, 1800

**Published:** 1800-03

**Authors:** 


### STATE OF DISEASES IN LONDON.

*Account of Diſeaſes in an Eaſtern Diſtrict from the 20th of January to the 20th of February, 1800.*

| | No. of Caſes. | | No. of Caſes |
|---|---|---|---|
| ACUTE DISEASES. | | Pleuriſy - - - - | 3 |
| Typhus Mitior - - | 4 | Catarrh - - - - | 5 |
| Peripneumonia Notha - | 10 | Acute Rheumatiſm - - | 3 |

CHRONIC

| | No. of Caſes. |
|---|---|
| CHRONIC DISEASES. | |
| Cough - - - | 19 |
| Dyſpnœa - - - | 10 |
| Cough with Dyſpnœa - | 23 |
| Paraphonia - - | 2 |
| Phthiſis Pulmonalis - | 4 |
| Hæmoptoe - - - | 3 |
| Pleurodyne - - - | 2 |
| Hydrothorax - - | 4 |
| Palpitatio - - - | 3 |
| Gaſtrodynia - - | 3 |
| Nauſea - - - | 2 |
| Vomitus - - - | 3 |
| Dyſpepſia - - | 6 |
| Hypochondriaſis - | 3 |
| Diarrhœa - - | 6 |
| Enterodynia - - | 4 |
| Hæmorrhois - - - | 3 |
| Hepatitis Chronica - | 1 |
| Enureſis - - - | 1 |

| | No. of Caſes. |
|---|---|
| Menorrhagia - - | 4 |
| Amenorrhœa - - | 5 |
| Fluor Albus - - - | 4 |
| Aſcites - - - | 3 |
| Anaſarca - - - | 4 |
| Hemiplegia - - | 2 |
| Epilepſy - - - | 1 |
| Hyſteria - - - - | 4 |
| Scrophula - - - | 3 |
| Chronic Rheumatiſm - | 20 |
| PUERPERAL DISEASES. | |
| Low Fever - - | 3 |
| Menorrhagia lochialis - | 5 |
| Maſtrodynia - - | 7 |
| INFANTILE DISEASES. | |
| Hooping Cough - - | 3 |
| Ophthalmia - - - | 2 |
| Convulſio - - - | 1 |
| Dentition - - - | 3 |
| Diarrhœa - - - | 4 |

The great ſeverity of the cold, and the long continuance of the eaſt and north-eaſterly winds, have occaſioned the continuance or return of pneumonic complaints.

The number of coughs and colds has been increaſed, and the ſymptoms in many caſes have been aggravated by the ſtate of the weather. The number of contagious diſeaſes has, however, been diminiſhed. The caſes of chronic rheumatiſm have increaſed in number, as appears by the liſt; and many of theſe have proved very obſtinate.

